# Internet-Based and Mobile-Based General Practice: Cross-Sectional Survey

**DOI:** 10.2196/jmir.8378

**Published:** 2018-09-25

**Authors:** Yan Qiu, Ying Liu, Wen Ren, Yunqing Qiu, Jingjing Ren

**Affiliations:** 1 The First Affiliated Hospital Zhejiang University Hangzhou China

**Keywords:** general practice, mHealth, mobile phone, satisfaction, health service, internet hospital

## Abstract

**Background:**

Globally, mHealth is increasing as a promising technology for promoting the quality of health care. Thus, a growing number of internet hospitals have been established in China to avail all its advantages. However, no study has investigated the service scope and patient satisfaction of the internet hospital to date.

**Objective:**

The objective of our study was to explore the features of outpatients in general practice, the disease information, and the satisfaction through an internet rating site.

**Methods:**

We collected data from the internet hospital of the First Affiliated Hospital, Zhejiang University between February 2016 and February 2017. Patients visited Web-based clinic via a computer or smartphone. The data included patients’ demographic characteristics, disease information, and patients’ comments.

**Results:**

We enrolled 715 patients with 365 health-related problems. All health conditions involved diseases ranging from internal medicine, surgery, gynecology and obstetrics, pediatrics, dermatology, ophthalmology, stomatology to emergency. Among them, 63.1% patients (451/715) visited traditional hospitals for further management, 25.3% (181/715) had prescriptions, laboratory, or imaging examination appointment, 1% (9/715) used emergency service, and 10% (74/715) needed routine follow-up. All patients received health education. Almost all patients gave positive feedback and 4-5-star rating.

**Conclusions:**

The internet hospital is suitable for all health conditions with high satisfaction only when patients have the access to internet via a computer or smartphone.

## Introduction

According to the World Health Organization, the global shortfall in health care workers will reach 12.9 million by 2035 and there exists a critical shortfall in most developing countries [1], increasing the burden on these countries’ health systems. The condition in China is more serious with the shortage of, at least, 161,000 general practitioners, 200,000 pediatricians, and 40,000 psychiatrists [2]. In addition, the national sixth census has reported that approximately 1.3 billion people live in China. All of these make it difficult for Chinese patients to see a doctor. Furthermore, because patients in China are free to choose a medical institution and a doctor, the majority of patients like to go to big-city, top-flight hospitals even for mild conditions, exacerbating the overload of health care workers [3,4]. With the rapid growth of the internet and the increasing use of the internet and mobile devices, global internet medical services have emerged.

The internet hospital is a new approach to provide health services, outpatient service in particular, through the internet technology [4,5]. Patients could use a smartphone app or Web to consult with qualified doctors at home, office, or other places as long as they have access to the internet. Doctors take patients’ history through a chatting platform designed for the internet hospital, and patients describe their condition and upload the relevant information, such as images and laboratory results, to the doctor through the smartphone app or Web. To date, studies have reported the characteristics of China’s internet hospitals, the health service capacity, and negative comments [5,6]; however, the spectrum and the satisfaction have not been involved. In recent years, patient rating sites have gained more attention for their function to measure the health care quality [7-9]. Moreover, its value for other patients looking for health care providers has been widely identified in the United States, Germany, and the United Kingdom [10]; however, research on patient rating sites in China remains sparse.

This study aims to identify who is suitable for the Web-based consultation via collection of outpatient characteristics and satisfaction of the internet hospital and clarify what kind of health services could be provided.

## Methods

### Internet Hospital of the First Affiliated Hospital, Zhejiang University

**Figure 1:**
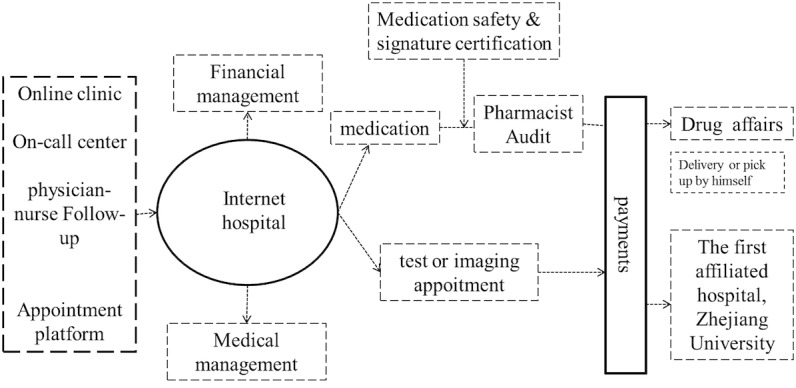
The flowchart of Web-based health service.

The internet hospital of the First Affiliated Hospital, Zhejiang University was put into use on February 16, 2016. At the beginning, 12 departments provided Web-based health services; of these, the general practice department bears nearly 30% Web-based workload. Figure 1 present the work flowchart [11]. The internet hospital provides outpatients with health services in the following 6 aspects: Web-based clinic; laboratory and imaging examination appointment; prescription; routine follow-up; various payments; and referral services. Based on Xie et al [5], the consulting methods included video chat, voice chat, telephone, image, and message. The details of the consultation are provided in a study conducted by Tu et al [4]. Regarding the laboratory and imaging examination appointment, patients could finish the laboratory and imaging examination in 1 day and earlier than the examination booked offline. The prescription is checked by the pharmacist with electronic signature (CA) and then delivered to or fetched by patients from the hospital or pharmacy. Of course, examination and prescription were provided after the fee was paid using many payment methods including Web-based (eg, Alipay and Apple pay), medicare, self-help machine, and others.

### Data Collection

Data were collected from the electronic record of the general practice department of the internet hospital between February 2016 and February 2017. Patients logged in the internet hospital with their computer or mobile phone. Overall, 715 consultations were provided.

The collected data included patients’ gender, age, region, consulting device, chief complaints, diagnose, management, and the satisfaction. The satisfaction was assessed through a specialized internet rating site, allowing patients to rate their experiences (1-5, 5 being the best) and freely express their comments with health care providers and the internet hospital. Furthermore, the satisfaction was extracted from all comments.

The disease diagnosis was encoded according to the International Classification of Diseases. The Institutional Review Board approval was obtained from the Research Ethics Committee of the First Affiliated Hospital College of Medicine, Zhejiang University (N2017-690).

### Statistical Analysis

Statistical analysis was performed using SPSS 19.0 (SPSS Inc., IL, USA) and Excel 2010 (Microsoft Corp., Redmond, WA, USA). The enumeration data are expressed in percentage, and the measurement data are expressed as mean (SD).

## Results

### Patients’ Characteristics

As shown in Table 1, 364 patients were men and 351 patients were women; the sex ratio was 1:0.96 with the age range from 3 months to 83 years and mean age, 36.81 (14.28) years. Most patients were from the Zhejiang Province with 29.9% (214/715) from Hangzhou city. Regarding the tools used, 42.9% (307/715) used the internet and 57.1% (408/715) used mobile phones.

### Information of Diseases on the Internet Hospital

We assessed 365 health-related problems on the internet hospital involving almost all departments, of which diseases belonging to internal medicine accounted for more than a half (53.6%). Table 2 shows that the former 5 symptoms were diseases of the musculoskeletal 12.8% (91/715), digestive 11.4% (81/715), reproductive 9.3% (66/715), urinary 7.7% (55/715), and endocrine 7.2% (51/715) systems. Table 3 shows that the number of patients who needed a referral to an offline clinic was 451, who had the medication or test appointment was 181, who need emergency services was 9, and who need follow-up was 74. All the patients had received health education.

### Satisfaction Analysis

**Figure 2:**
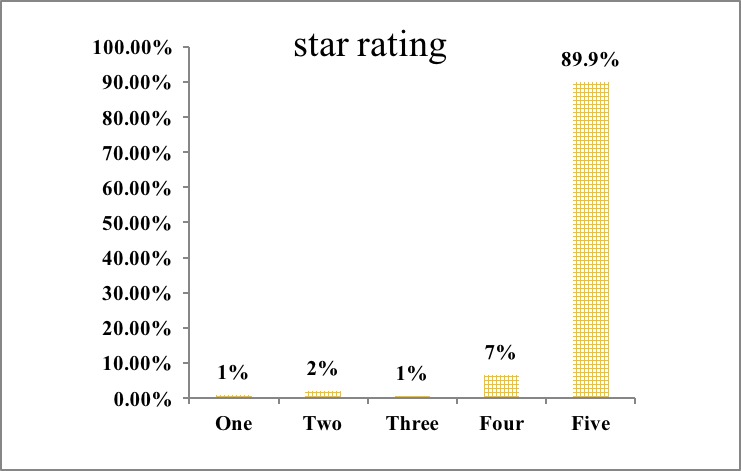
Patients’ star rating (1 to 5 stars: worst to best).

In this study, 384 patients gave comments on the internet rating site, as seen in Multimedia Appendix 1; of these, 375 were satisfied with the Web-based clinic (satisfaction rate, 97.7%). Figure 2 shows that 89.9% (345/384) of participants rated 5-star. Regarding the contents (Table 4), the most common positive comments of the internet hospital and general practitioner were “quick and convenient, efficient, powerful function of the internet hospital, professional, full of enthusiasm, responsible, full of patience and meticulousness, gentle, friendly, and so on.”

Of patients who were unsatisfied with Web-based service, the comments were waiting too long, unable to solve the concerns, misuse of medical terminology, and impatient.

**Table 1:** Patients’ characteristics (N=715).

Baseline variables		Patients, n (%)
Hangzhou		214 (29.9)
**Sex**		
	Male	364 (50.9)
	Female	351 (49.1)
**Age**		
	<18 y	17 (2.4)
	18-59 y	648 (90.6)
	60-69 y	27 (3.8)
	≥70 y	23 (3.2)
**Tools used**		
	Internet user	307 (42.9)
	Mobile phone user	408 (57.1)

**Table 2:** Symptoms classification in different system pattern (N=715).

System	Patients, n (%)
Musculoskeletal	91 (12.8)
Digestive	81 (11.4)
Reproductive	66 (9.3)
Urinary	55 (7.7)
Endocrine	51 (7.2)

**Table 3:** The percentage of different general practitioners’ management (N=715).

Management	Patients, n (%)
Emergence	9 (1.3)
Referral	451 (63.3)
Further follow-up	74 (10.4)
Medication or test appointment	181 (25.4)

**Table 4:** Patient’s assessment according to the contents (n=384).

Contents		Patients, n (%)
**Doctor**		
	Of good attitude, full of patience and meticulousness, gentle and friendly	40 (10.4)
	Of good responsibility	5 (1.3)
	Professional	7 (1.8)
	Competent	24 (6.3)
	Good answers	4 (1.0)
**Internet hospital**		
	Powerful	8 (2.1)
	Quick and convenient	51 (13.3)
**Brief comments**		
	Satisfaction	32 (8.3)
	Ok, good, very good, and thanks	141 (36.7)
	Bad	6 (1.6)
	No comments	66 (17.2)

## Discussion

### Principal Findings

This study focused on the consultation spectrum and the satisfaction of the internet hospital and analyzed the data from the general practice department. Patients with all health-related conditions could seek health services in the internet hospital, and the satisfaction was high.

In this study, we observed that the majority of outpatients were male and adults. Among patients who are children and older adults, most sought the Web-based health services with the help of families because they could not operate a smartphone or computer. Hence, with regard to these patients, most tended to choose traditional hospitals, resulting in the small number of Web-based patients in the study. In addition, other reasons were as follows. First, not many know about the existence of the internet hospital of the First Affiliated Hospital, Zhejiang University, which we believe could be changed through propaganda and promotional activities, for instance, we provided free consultations over one year. Second, no explicit protocol exists as to what symptoms could be solved online. Thus, some people might be confused and hesitate to see the Web-based doctor. In this study, we found that all health-related problems could be consulted online initially, which is consistent with the role of general practitioners. Because general practice is the primary point of access to health care services, general practitioners play a role in diagnosing and treating illness within the community, promoting better health, preventing disease, certifying disease, monitoring chronic disease, and referring patients requiring specialist services [12].

In recent years, the remarkable proliferation of mobile technology has facilitated health systems through interventions that increase both the coverage and quality. In addition, the mHealth awareness among the general population is growing. Among 715 Web-based consultations, >50% patients used mobile phones to access the health service; this finding is similar to that obtained by Reem et al [13]. Because most patients were not from Hangzhou city, the internet hospital is beneficial and available to patients who own a mobile or computer despite not being residents of neighboring areas. Certainly, this is attributed to the advantages of mHealth, easy and flexible to access without local or temporal boundaries.

In addition, we found higher satisfaction with Web-based consultation, which could be attributed to 2 possible reasons. First, people in China tend to trust “big hospitals,” because they tend to go to high-level hospitals even for mild symptoms [3,4]. In addition, all Web-based doctors are highly qualified physicians in the First Affiliated Hospital, Zhejiang University, one of the best tertiary hospitals in Zhejiang Province; they have >3-year experience without complaint records. Second, the internet hospital here could provide a prescription with delivery service and appointment services earlier than the traditional ones. It is unlike other cloud hospitals in which physicians could just provide a recommendation because they may come from a hospital in this city and the patient residing in another city. Therefore, it is inconvenient to provide earlier laboratory or imaging service. This study, indeed, clarified the point. Nevertheless, most individuals favor the internet hospital, implying that mHealth might play a great role in the field of medicine in the future; these results were similar to those obtained by Reem et al [13]. Third, because the number of Web-based outpatients was smaller than the offline, physicians had more time for each outpatient, making patients feel better than the crowded traditional clinics.

### Limitations

Although internet- and mobile-based hospitals could alleviate the dilemma of difficulty to see a doctor in the developing countries and cater for most residents, limitations persist. First, there are problems with the establishment of the internet hospital. To date, no legal support exists for the establishment of the internet hospital and practice of physicians. In addition, there are no incision and quality-control standards and no safety protection system. All of these should be solved along with the efforts of the government. Luckily, the National Health and Family Planning Commission of the People’s Republic of China have realized the problem and started planning the regulations. Second, because the rating site had no mandatory requirements for the comment and comment list, no data exist as to whether they would like to revisit the Web-based clinic. Hence, the data could be obtained through further analysis for consultation times per person.

### Conclusions

When individuals have access to the internet with a computer or smartphone, internet- and mobile-based Web-based practice is feasible and convenient for almost all health-related conditions. Although the satisfaction was high, the internet rating site should be put to better use to improve Web-based health services.

## Appendix

Multimedia Appendix 1 An example of positive comments from the rating site (access from online hospital of the first affiliated hospital, Zhejiang University).
