# Supplementary figures and images for: Internet-Based and Mobile-Based General Practice: Cross-Sectional Survey
Source: J Med Internet Res. 2018 Sep 25;20(9):e266. doi: 10.2196/jmir.8378 (PMC6300040; doi:10.2196/jmir.8378)

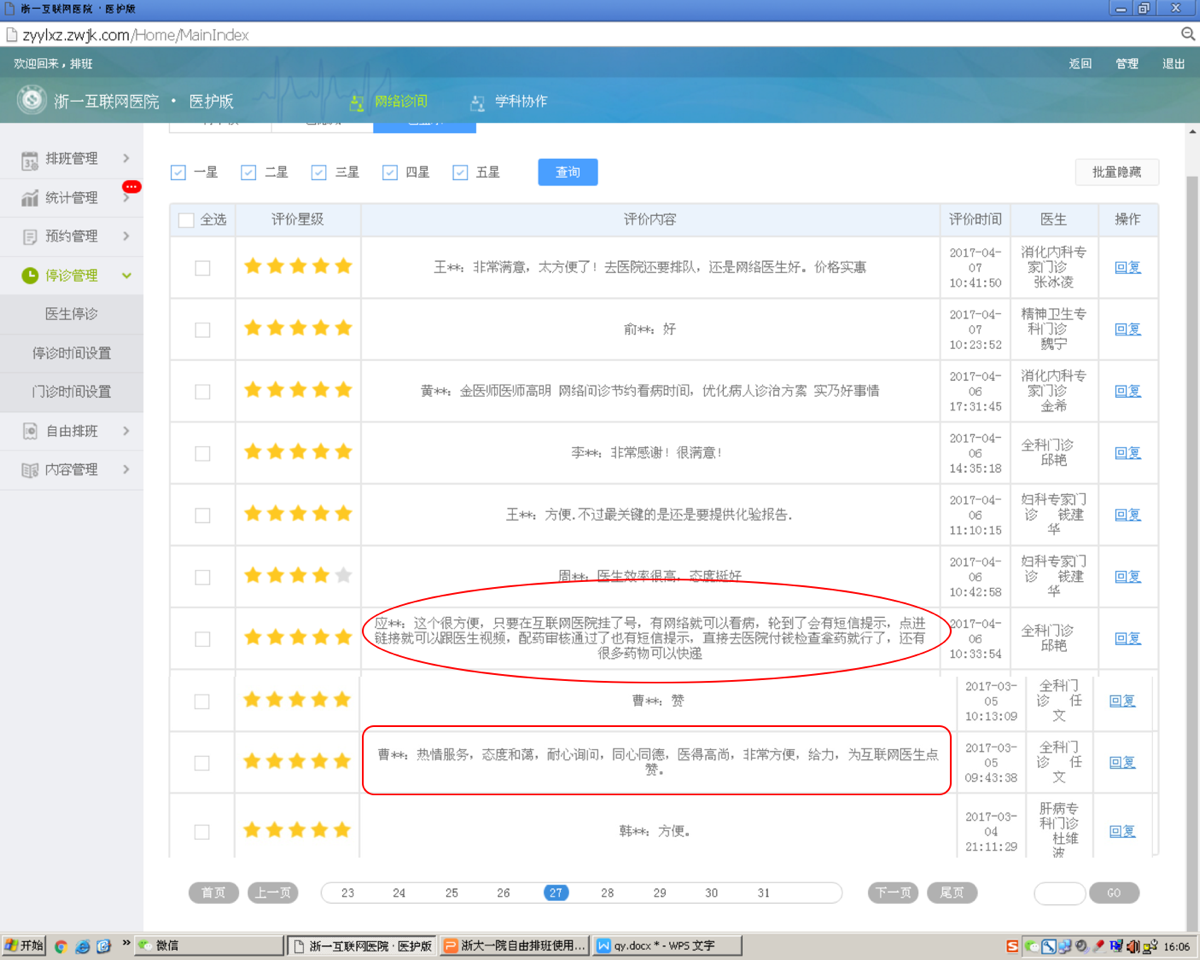

Supplement: Multimedia Appendix 1 [file jmir_v20i9e266_app1.png]
